# Running in the wheel: Defining individual severity levels in mice

**DOI:** 10.1371/journal.pbio.2006159

**Published:** 2018-10-18

**Authors:** Christine Häger, Lydia M. Keubler, Steven R. Talbot, Svenja Biernot, Nora Weegh, Stephanie Buchheister, Manuela Buettner, Silke Glage, André Bleich

**Affiliations:** Institute for Laboratory Animal Science, Hannover Medical School, Hannover, Germany; University of Amsterdam, Netherlands

## Abstract

The fine-scale grading of the severity experienced by animals used in research constitutes a key element of the 3Rs (replace, reduce, and refine) principles and a legal requirement in the European Union Directive 2010/63/EU. Particularly, the exact assessment of all signs of pain, suffering, and distress experienced by laboratory animals represents a prerequisite to develop refinement strategies. However, minimal and noninvasive methods for an evidence-based severity assessment are scarce. Therefore, we investigated whether voluntary wheel running (VWR) provides an observer-independent behaviour-centred approach to grade severity experienced by C57BL/6J mice undergoing various treatments. In a mouse model of chemically induced acute colitis, VWR behaviour was directly related to colitis severity, whereas clinical scoring did not sensitively reflect severity but rather indicated marginal signs of compromised welfare. Unsupervised k-means algorithm–based cluster analysis of body weight and VWR data enabled the discrimination of cluster borders and distinct levels of severity. The validity of the cluster analysis was affirmed in a mouse model of acute restraint stress. This method was also applicable to uncover and grade the impact of serial blood sampling on the animal’s welfare, underlined by increased histological scores in the colitis model. To reflect the entirety of severity in a multidimensional model, the presented approach may have to be calibrated and validated in other animal models requiring the integration of further parameters. In this experimental set up, however, the automated assessment of an emotional/motivational driven behaviour and subsequent integration of the data into a mathematical model enabled unbiased individual severity grading in laboratory mice, thereby providing an essential contribution to the 3Rs principles.

## Author summary

Animal-based biomedical research is often accompanied by experience of discomfort or pain by the animal. Recognition of disturbed animal welfare is mandatory, and the classification and assessment of its severity is a crucial part of the legislative framework in the European Union (EU). In the present study, we analysed voluntary wheel running (VWR) behaviour as a measure of compromised welfare in a mouse colitis model. Unsupervised mathematical clustering of clinical and VWR data enabled us to allocate and classify severity levels. This cluster model was verified using VWR data from a restraint stress model and allowed us to uncover the impact of routine experimental procedures on these mice. We propose that clustering of VWR behaviour provides a useful method for assessing the severity level of experimental procedures conducted on mice.

## Introduction

The 3Rs (replace, reduce, and refine) principles [1] provides a fundamental ethical and statutory framework to embed animal welfare into biomedical research. Scientists, laboratory animal science associations, journals, and countries around the globe committed themselves to this principle. With respect to the refinement approach, the fine-scale grading of severity in laboratory animals undergoing scientific procedures is indispensable to improve welfare and minimize suffering. Accordingly, the assessment of severity experienced by laboratory animals has become a prerequisite for the project authorization process in the revision of the European Union (EU) Directive on the protection of animals used for scientific purposes [2]. In particular, every procedure performed on laboratory animals has to be allocated prospectively and retrospectively to the categories ‘non-recovery’, ‘mild’, ‘moderate’, and ‘severe’ with regard to the respective pain, suffering, distress, or lasting harm to the animals (Article 38, 39, 54 and Annex VIII of Directive 2010/63/EU). However, tools to assign an experimental procedure to a specific severity level are scarce and abilities to assess the entire spectrum of severity are limited [3,4]. Particularly, there is a lack of objective, standardized parameters that are routinely applicable and non- or minimally invasive. Therefore, the development of evidence-based techniques and scales grading severity in laboratory animals is crucial not only regarding the legal obligations and the demand for standardized high-quality data but also with regard to the ethical justification of animal-based research [3].

Voluntary wheel running (VWR), an elective behaviour in wild mice [5], has been scientifically assessed as early as 1898 [6] and demonstrated to differ between mouse strains and gender [7–9]. The effect of VWR has been investigated in numerous studies regarding inactivity-related diseases such as obesity [10], cardiovascular disease [11], and type II diabetes [12]. It also served as an outcome measure to monitor motor function deficits [13] and circadian rhythm [14]. Furthermore, VWR has been utilized to determine pain-related mobility impairment in a study investigating hind paw inflammation [15] and to characterize a chronic pancreatitis model associated with persistent abdominal pain [16].

As VWR has been shown to be biologically distinct from general activity and is associated with neuronal systems allocated to stress response, mood, and reward [17], it may reflect the motivational, emotional, and cognitive state of animals. Therefore, we hypothesized that VWR serves as a tool to assess and classify severity of a multidimensional nature in laboratory mice. To evaluate VWR behaviour as a measure of treatment-associated discomfort, mice underwent either finely graduated acute intestinal inflammation or restraint stress and/or different sampling procedures. Subsequent k-means algorithm-based cluster analysis of VWR and body weight data revealed distinct severity levels, providing a novel approach for objective individualized severity grading in laboratory mice.

## Results

### Dose-dependent determination of colitis-induced severity progression by monitoring of VWR behaviour

**Fig 1:**
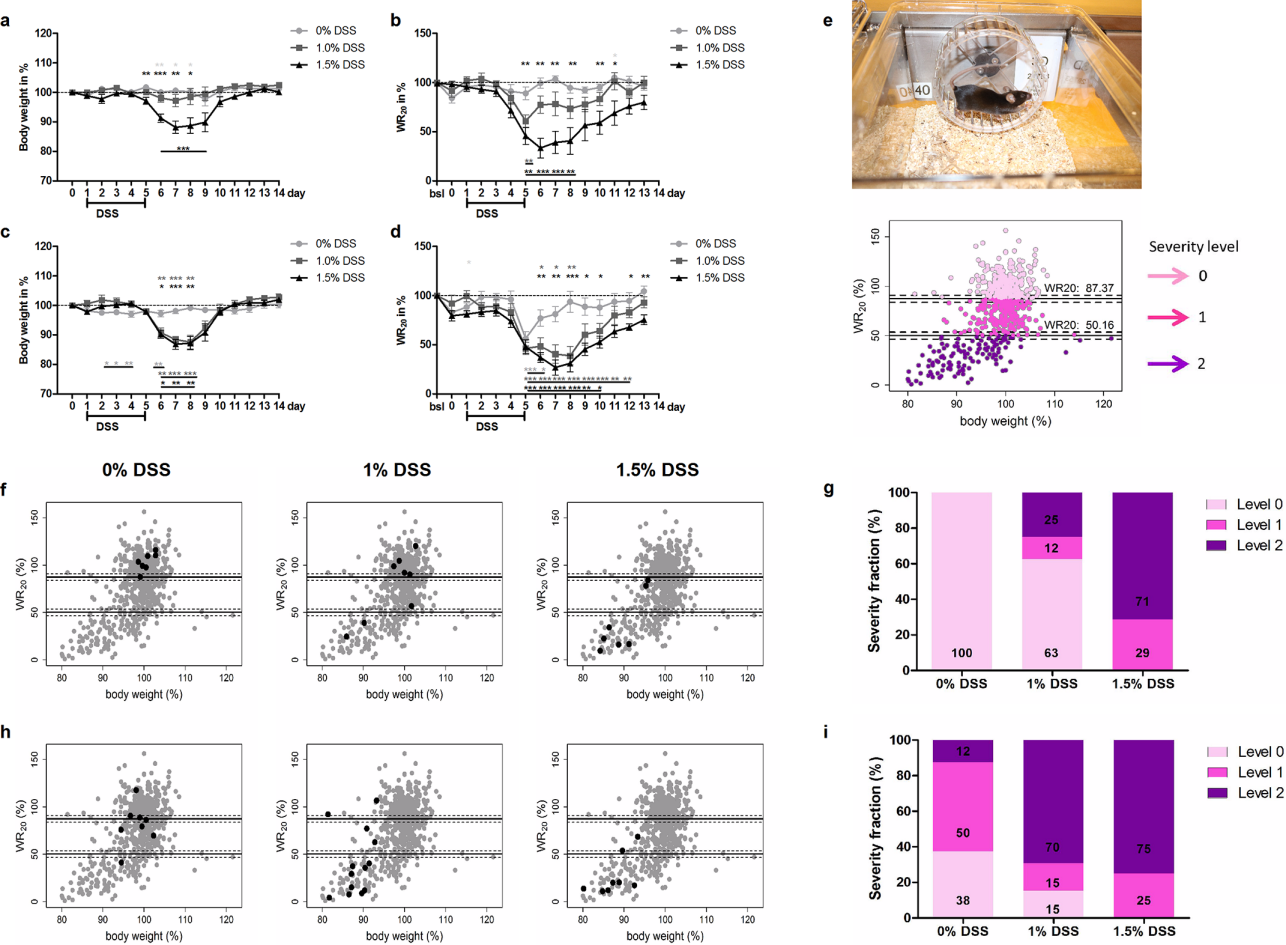
Assessment of severity during acute intestinal inflammation. (a) Determination of body weight (% change from baseline) and (b) WR_20_ (percent change from baseline) in mice receiving 0%, 1%, or 1.5% DSS. (c) Body weight and (d) WR_20_ over time in mice receiving 0%, 1%, or 1.5% DSS and additionally undergoing facial vein phlebotomy on d 0, d 5, and d 14. All mice of (a–d) underwent faecal sampling. For *n* values see S1 Table. *P < 0.05, **P < 0.01, and ***P < 0.001; colours indicate comparison between respective groups: medium grey between 0% and 1%, black between 0% and 1.5%, and light grey between 1% and 1.5% (one-way ANOVA, subsequent Tukey posthoc test or Kruskal–Wallis test followed by Dunn’s multiple comparison test); underlined asterisks indicate the comparison to baseline levels within a group (repeated measure ANOVA, subsequent Dunnett’s posthoc test or Friedman test followed by Dunn’s multiple comparison test). (e) B6 mouse demonstrating VWR behaviour in a running wheel; WR_20_ of all mice (a–d) plotted against body weight in k-means cluster analysis with cluster borders (solid lines) and 95% confidence borders (dashed lines). (f) Cluster analysis as in (e), DSS-treated mice at d 7 individually highlighted in black; (g) the corresponding calculation of severity fractions. (h) Cluster analysis as in (e), DSS-treated mice at d 7 that were submitted to facial vein phlebotomy individually highlighted in black; (i) the corresponding calculation of severity fractions. The underlying numerical data of each figure panel are provided in the respective excel sheet of S1 Data; underlying numerical data of Fig 1F–1I are provided in the corresponding sheet of Fig 1E. B6, C57BL/6J; DSS, dextran sulfate sodium; VWR, voluntary wheel running; WR_20_, wheel rotations during 20 hours/day.

VWR was monitored in C57BL/6J (B6) mice that were treated with either 0%, 1%, or 1.5% dextran sulfate sodium (DSS) to induce acute intestinal inflammation. Furthermore, VWR was monitored in DSS-treated B6 mice that additionally underwent facial vein phlebotomy (for an overview of groups and *n* values see S1 Table). All mice were single housed in cages supplemented with a running wheel (Revolyzer 3TS system, software DASY Lab 11.0) that allowed monitoring of wheel rotations (WR_20_) and maximum velocity (Vmax_20_) of 20 hours/day. During the 14-day (d) adaptation phase, WR_20_ and Vmax_20_ increased continuously, reaching a consistent plateau after 9 days (S1 Fig). Mean WR_20_ and Vmax_20_ of the last 3 days of the respective adaption phases served as the baseline to calculate the relative change in %. Subsequent experimental procedures comprised faecal sampling (all groups, Fig 1A–1D); blood sampling (selected groups, Fig 1C and 1D) on d 0, d 5, and d 14; DSS treatment (d 1–d 5), and necropsy (d 14) (see S1 Table). Mice were monitored daily by clinical scoring and weighing.

Significant weight loss up to 21.6% (mean 11.9% ±1.9% SEM) on d 7 was observed in mice treated with 1.5% DSS but not 1% DSS, compared to controls (d 7, Kruskal–Wallis test statistic: 12.22, df = 2; Dunn’s test *P* < 0.01, Fig 1A). Accordingly, clinical scoring was solely but merely marginally increased in the 1.5% treatment group (S2A Fig). In contrast, WR_20_ was reduced in both treatment groups, rendering the monitoring of VWR behaviour more sensitive than clinical scoring in determining disease progression in a dose-dependent manner (d 7, Kruskal–Wallis test statistic: 11.97, df = 2; Dunn’s test *P* < 0.01 for 1.5% versus 0% DSS group, Fig 1B). Vmax_20_ was reduced solely in mice treated with 1.5% DSS (S3A Fig). Next, serial blood sampling by facial vein phlebotomy, a sampling procedure frequently applied in animal-based research, was performed on d 0, d 5, and d 14 in DSS-treated and control mice (see S1 Table for groups and *n* values). Unexpectedly, WR_20_ was significantly reduced in DSS-treated and control mice after blood sampling (d 5, repeated measure ANOVA F_(6.221)_ = 21.47 [0% DSS], F_(19.84)_ = 21.90 [1% DSS]; Dunnett’s tests *P* < 0.001 compared to baseline; Friedman test statistic: 83.05 [1.5% DSS]; Dunn’s test *P* < 0.001 compared to baseline, Fig 1D). Additionally, blood sampling not only impacted VWR behaviour but also aggravated colitis progression, as 1% DSS-treated mice now displayed a similar course of body weight loss, WR_20_, and Vmax_20_ as 1.5% DSS-treated mice (Fig 1C and 1D and S3B Fig). The aggravated condition was not detected by clinical scoring (S2B Fig) but was corroborated by histological analysis (S4 Fig).

### Demarcation of individual severity levels by k-means algorithm-based cluster analysis

To enable unbiased severity allocation, k-means cluster analysis based on behavioural data sets (VWR performance) and clinical data sets (body weight measurements) derived from all DSS-treated and respective control mice, including their baseline values, was determined to be suitable. Interestingly, an optimal cluster size of three clusters was obtained by scree plot analysis as well as calculation of the Bayesian information criterion (S5A and S5B Fig). Cluster stability was monitored by permutation analysis. Cluster borders were calculated to be WR_20_ = 87.37% and WR_20_ = 50.16%, with 95% confidence borders (83.75; 90.39) and (46.43; 53.57), respectively (Fig 1E and S5C Fig). Accordingly, three severity categories were classified as ‘severity level 0, 1, and 2’, respectively (depicted in Fig 1E). Exemplary highlighting of mice at d 7 demonstrated that all of the control mice (0% DSS) were allocated to severity level 0, whereas the distribution of 1% and 1.5% DSS-treated mice shifted toward severity levels 1 and 2 (Fig 1F). Calculation of the percental proportion of mice assigned to a particular severity category (‘severity fraction’) for each treatment regime revealed that 100% of the control mice were allocated to severity level 0 and none were assigned to severity level 2 (Fig 1G). However, this was reversed in 1.5% DSS-treated mice, as 71% of mice were allocated to severity level 2 and none to severity level 0. Highlighting of 1% and 1.5% DSS-treated mice that additionally underwent facial vein phlebotomy revealed a shift in the distribution pattern toward severity levels 1 and 2, respectively (compare Fig 1F and Fig 1H), further corroborating an aggravated condition due to this routine blood sampling procedure. Merely 38% of control mice (0% DSS) were allocated to severity level 0 but 12% to severity level 2 following routine blood sampling (Fig 1I).

### Derivation of distinct severity levels in a mouse model of restraint stress affirms applicability of VWR behaviour–based k-means clustering for individual severity grading

**Fig 2:**
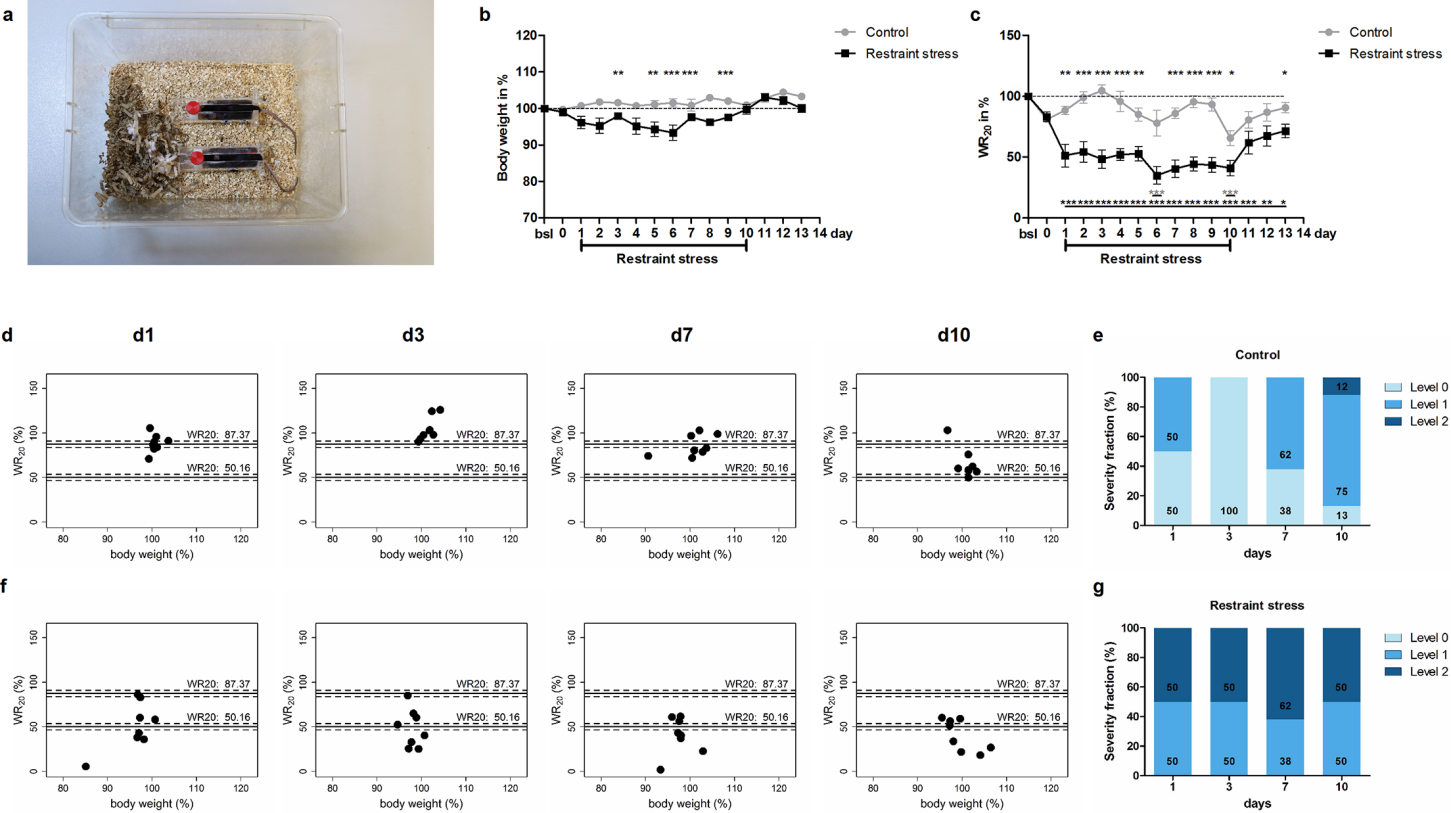
Assessment of severity during restraint stress. (a) Restrained mice in their home cage. (b) Determination of body weight (*n* = 8) and (c) WR_20_ (*n* = 8) in control and restrained mice, all of which underwent faecal sampling (d 0, d 7, d 10). For groups and *n* values see also S1 Table. *P < 0.05, **P < 0.01, and ***P < 0.001, comparison between groups (Mann–Whitney or unpaired *t* test with Welch’s correction in case of unequal variance); underlined asterisks indicate the comparison to baseline levels within a group (Friedman test followed by Dunn’s multiple comparison test). Incorporation of restraint stress data at d 1, d 3, d 7, and d 10 into the cluster model; (d) control mice with (e) the corresponding calculation of severity fractions; and (f) restraint-stressed mice with (g) the corresponding calculation of severity fractions. The underlying numerical data of each figure panel are provided in the respective excel sheet of S1 Data; underlying numerical data of Fig 2D–2G are provided in the corresponding sheet of Fig 1E.

As a next step, the applicability of the cluster model as a tool for severity categorization was tested in mice submitted to restraint stress. In this model, mice were immobilized using restraint tubes for 1 hour from d 1 to d 10. These and respective control mice underwent faecal sampling on d 0, d 7, and d 10. Clinical scoring and body weight were merely marginally altered in restraint-stressed mice (S2C Fig and Fig 2B). However, WR_20_ was significantly reduced to approximately 50% of baseline performance from d 1 to d 10 in restraint stressed mice (repeated measure ANOVA F_(7.15)_ = 7.337; Dunnett’s test *P* < 0.05–0.001, Fig 2C). Interestingly, a drop in WR_20_ was also observed on days of faecal sampling (d 0, d 7, and d 10) in both control and restraint-stressed mice (Fig 2C). Reduction of Vmax_20_ in restraint-stressed mice was less pronounced than reduction of WR_20_ (S3 Fig). Next, these data were tested in the cluster model, revealing an equal distribution of control mice into severity level 0 and 1 on d 1 (Fig 2D and 2E), which might be attributed to the impact of faecal sampling on d 0. This effect of the sampling procedure was also discernible on d 7 and d 10, whereas all control mice on d 3 were categorized into severity level 0 (Fig 2D and 2E). However, the distribution pattern in mice undergoing restraint stress markedly shifted into severity levels 1 and 2, with up to 62% of restraint-stressed mice allocated to severity level 2 on d 7 (Fig 2F and 2G).

## Discussion

VWR behaviour has been experimentally utilized as both a variable to detect its effect on metabolic and cardiovascular models [10–12] as well as an index for pain-related or neurological impairment [15,16,18]. It is a complex behaviour and has recently been used in mouse models of motor deficits to identify new factors delineating motor function previously not detected in rotarod tests [19]. In addition, VWR has been demonstrated to alter neuronal circuity by induction of neurogenesis [20,21]. With regard to the induction of these neuroanatomical and physiological changes, VWR does not merely present a measure for general activity but may rather serve as a behavioural readout, as it also has been demonstrated to decrease anxiety- and depression-like behaviours [22,23]. Moreover, VWR represents a strongly motivated behaviour and consequently reinforces learning capacities such as operant conditioning to obtain access to a running wheel in rodents [24]. Additionally, this reinforcing effect has been demonstrated to exceed the positive reinforcing effect of addictive drugs [25]. We therefore speculated that VWR behaviour may not only be utilized as an indicator for pain-related mobility impairment but rather as a measure to reflect various facets of severity in an emotional/motivational behaviour-centred approach. To our knowledge, it has not yet been addressed whether VWR behaviour can be utilized to assess severity conditions in laboratory mice. Therefore, VWR behaviour was tested in the present study as an indicator of treatment-associated discomfort during acute intestinal inflammation, acute stress, and sampling procedures and was demonstrated to serve as an early and sensitive indicator of compromised welfare in these conditions.

Chemical induction of intestinal inflammation via graded doses of DSS resulted in a dose-dependent reduction in VWR behaviour in 1% and 1.5% DSS-treated mice (Fig 1B). In contrast, increased clinical scores and reduced body weights appeared delayed and occurred only in the 1.5% treatment group, suggesting that VWR is an earlier and more sensitive indicator of compromised welfare (Fig 1A and S2A Fig). Similarly, serial blood sampling by facial vein phlebotomy led to reduced VWR behaviour in both control and DSS-treated mice but was not discernible by clinical scoring (S2B Fig). In addition, and rather unexpectedly to this extent, aggravation of the course of colitis as reflected by increased histological scores and a greater reduction of body weight were also observed due to serial blood sampling (S4 Fig and Fig 1C and 1D). In a recent study, facial vein phlebotomy had the mildest effect on animal welfare when the impact of single sublingual vein puncture, tail vein puncture, retrobulbar plexus/sinus puncture, and facial vein puncture were compared [26]. In another study, tail tip amputation was identified as the least compromising procedure when compared to facial vein puncture and lateral tail vein incision [27]. Blood sampling is a common procedure in laboratory animal-based research and may not only have a potential impact on the animal with regard to compromised welfare but may also interfere with the research model of choice and the respective readouts. In the present study, the utilized blood-sampling routine was a complex procedure comprising routine handling, restraining, and the actual transfer of the animals in itself. Therefore, at this time, we cannot identify the most compromising act, and this needs to be addressed in future investigations.

VWR behaviour not only served as indicator of compromised welfare during acute colitis and serial blood sampling but also during acute stress. Immobilization stress led to an early (d 1) and substantial reduction of VWR behaviour but only resulted in a marginal increase in clinical scores and a slight reduction of body weight (S2C Fig and Fig 2B and 2C). Interestingly, another sampling procedure effect was detected as a drop in WR_20_ on days of faecal sampling in both control and restraint-stressed mice (Fig 2C).

As a consequence, the potential interference of sampling procedures should be taken into consideration in study design and experimental set up. This also applies to other factors that have been demonstrated to induce stress and anxiety in mice, like the applied handling method [28,29] or the presence of male experimenters [30]. In the present study, all animals were handled identically and by females.

Regarding the suitability of VWR behaviour as an indicator of compromised welfare, monitoring of WR_20_ proved a more suitable parameter to detect treatment-associated differences than changes in running velocity (Vmax_20_, S3 Fig), which were not as pronounced than those observed in WR_20_ (Fig 1 and Fig 2).

K-means algorithm-based cluster analysis [31] has served as a tool for a variety of research purposes, e.g., neuronal classification [32], differentiation of cell populations [33], and distinction of necrosis from viable tissue via MRI [34]. Cluster analysis has also been utilized for gene expression analysis and associated disease outcomes [35] and recently to classify plantar pressure distribution, which is critical for the prevention and/or treatment of the diabetic foot [36]. The DSS-induced acute mouse model of colitis represents a multidimensional model with various inherent features of severity such as anxiety/depression and pain [37,38]. Therefore, we considered data derived from this model as an optimal ‘training data set.’ Consequently, VWR and body weight as objective, observer independent data were used to develop a cluster model. Cluster borders were calculated at WR_20_ = 87.37% and WR_20_ = 50.16%, defining severity levels 0, 1, and 2 (Fig 1E and S5C Fig). By identification of these three categories, an evidence-based assessment into ‘no’, ‘low’, or ‘moderate’ severity grades may be possible. The applicability of the cluster model was successfully tested in this study by introducing ‘unknown’ data from the mouse model of acute stress. Here, restraint-stressed mice were constantly allocated to severity level 1 or 2 over the duration of the restraint procedure (see Fig 2F and 2G). So far, experience- and consensus-based approaches for assessing severity in laboratory mice substantially rely on clinical score sheets. However, scoring may vary between observers [39], nuances of severity may not be detected, especially in prey animals, and standardisation in clinical scoring has been reported to be insufficient [40], underlining the need for observer-independent approaches. A long-established, relevant parameter is the change of body weight [41]. Here, a generally accepted criterion of a ‘severe’ condition is a body weight loss exceeding 20% that may lead to euthanasia [42], although it does not reflect body composition or model specific dynamics [43]. In this study, the majority of mice that reached up to 20% body weight loss (defined as a humane endpoint) were allocated to severity level 2, indicating compromised welfare according to cluster analysis of VWR behaviour (Fig 1E). However, during the analysis, we noticed mice with a substantial body weight loss but without decreased VWR behaviour that therefore clustered in severity level 0 (Fig 1). This clearly emphasizes that a combination of robust parameters is needed to reflect the actual severity experienced by an animal.

To obtain automated individual data sets, mice were single housed in the present study, which potentially represents another stressor. Nevertheless, mice were kept in clear open cages, facilitating visual and auditory contact for the duration of the experiments. In general, mice are recommended to be housed in groups to avoid social isolation and to maximize wellbeing [2], but several studies have demonstrated that single housing did not lead to increased stress markers compared to group housing [44–46]. Furthermore, in a study of postsurgical behaviour, no distinct negative effect was discernible in single-housed mice [47]. In addition, in a study of morphine withdrawal, the attenuation of the increase in thermal sensitivity was actually greater in single-housed mice with access to a running wheel than in group-housed mice without access to a wheel [48]. Meanwhile, novel wheel running systems that allow group housing whilst accomplishing the simultaneous monitoring of individual VWR performances are available and potentially applicable.

The categorization of severity has become a statutory requirement for the project authorization process in European legislation. As appropriate methods for severity assessment and classification are missing, the resulting gap between current regulations and scientific knowledge has to be filled. Our novel approach of unbiased individual severity grading enabled classification of independent models or stressors in B6 mice, which we made available as an online tool at https://calliope.shinyapps.io/severity_assessment/. Applicability to other mouse models and strains is probable but needs to be tested in future studies. This might require adaptation of the parameters to be involved in the assessment because of the multidimensional nature of severity as well as particularities of animal models and mouse strains. In conclusion, VWR behaviour served as a refinement tool in an easily implemented home-cage–based approach. It should therefore be considered in future studies as a parameter in animal welfare and severity assessment strategies to sensitively discriminate individual severity levels in mice.

## Materials and methods

### Ethics statement

This study was conducted in accordance with the German law for animal protection and the European Directive, 2010/63/EU. All experiments were approved and permitted by the Lower Saxony State Office for Consumer Protection and Food Safety (LAVES, license 15/1905).

### Mice and experimental set up

Ten–thirteen-week old female B6 mice were obtained from the Central Animal Facility (Hannover Medical School, Hannover, Germany). Routine health surveillance and microbiologic monitoring according to the Federation of European Laboratory Animal Associations recommendations did not reveal any evidence of infection with common murine pathogens [49,50]. Mice were maintained in a room with controlled environment (21°C–23°C; relative humidity 55% ± 5%; 14:10-hour light:dark cycle). Mice were housed in macrolon cages (360 cm^2^) with softwood granulates (poplar wood, AB 368P, AsBe-wood GmbH, Germany) and cleaned once per week. Pelleted diet (Altromin 1324, Lage, Germany) and autoclaved water were provided ad libitum. During the 2-week habituation to the room, animals were merely handled for cage cleaning.

For each experimental set up, a different cohort of mice was used (as specified in S1 Table). Sample size calculations were performed using the power analysis program G*Power 3.1 [51]. *N* values are given in S1 Table. Animals were then divided into treatment and control groups by applying a random selection procedure (drawing lots).

All mice of this study had access to running wheels. Prior to study initiation, a 2-week adaption phase to the running wheel was chosen as outlined below. In the cohorts, the experimental set up was as follows: animals were treated with DSS (0% [control], 1%, or 1.5%) from d 1 to d 5. In these mice, faecal sampling was performed on d 0, d 5, and d 14. Additional DSS-treated mice (0% [control], 1%, or 1.5%) underwent faecal sampling as well as phlebotomy on d 0, d 5, and d 14. Additional mice were used in the restraint stress model. In these groups, restraint stress was applied from d 1 to d 10. In these and respective control mice, faecal sampling was performed on d 0, d 7, and d 10.

Handling during experimental procedures was performed in reference to Sorge and colleagues solely by females [30]. Mice were handled by the tail, i.e., the mice were grasped by the base of the tail using the thumb and forefinger and then transported on the flat of the hand to support the body.

### VWR

Mice were single housed in home cages with free access to a running wheel (diameter of 11.5 cm, Revolyzer 3TS system, software DASY Lab 11.0 preclinics GmbH, Germany) that allowed automatic and undisturbed 20-hour monitoring of wheel rotations (WR_20_) and maximum velocity (Vmax_20_, referring to the maximal number of wheel rotations per minute recorded during the 20-hour period) from 12:00 PM to 08:00 AM daily, leaving a 4-hour interval for general maintenance and experimental procedures (depending on the cohort, e.g., weighing, phlebotomy, restraint stress). To determine the steady state running performance, an adaption phase of 14 days was chosen before subsequent experiments (see also S1 Fig). During the adaption phase the health status of the animals was monitored twice per week. All B6 mice started to run as soon as they were introduced into the cage supplemented with the running wheel. The peak time of running expectedly occurred during the dark phase. For subsequent WR_20_ and Vmax_20_ analysis, the mean of the last 3 days of the respective adaption phases were set as the baseline to calculate relative changes (%).

### Induction of DSS colitis

To fully control the onset, duration, and degree of intestinal inflammation for relating severity assessment parameters to the degree of colitis [52,53], an acute colitis model induced by DSS (mol wt 36,000–50,000; MP Biomedicals, Eschwege, Germany) was chosen. Mice of the respective cohorts (see also S1 Table) were exposed to 0% (control group), 1%, and 1.5% DSS in drinking water for 5 consecutive days (d 1–d 5) to induce a mild to moderate intestinal inflammation. Mice were weighed and monitored daily according to the clinical score described below. To prevent severe conditions, a body weight loss ≥ 20% was defined as a humane endpoint.

### Restraint stress

To induce acute stress mice were inserted into restraint tubes on 10 consecutive days (d 1–d 10) for 60 minutes (from 09:00 to 10:00 AM) and placed in empty housing cages during the restraint period. Restraint tubes (23-mm internal diameter, 93-mm length) consisted of clear acrylic glass with ventilation holes (8-mm diameter) and a whole length spanning 7-mm–wide opening along the upper side of the tube. The ends of the tube were sealed on one side by a piece of acrylic glass with a slot for the mouse tail and on the other end by a screwable solid plastic ring. Mice were able to rotate around their own axis but not to move horizontally.

### Clinical scoring

Clinical scoring was performed daily by the same person between 08:00 AM and 09:00 AM, as described recently [54] including the parameters stool consistency, posture, behaviour, and the appearance of eyes and fur. Clinical scoring constituted a base parameter mandatory for project authorization and was performed by an experienced veterinarian, which was not blinded to the treatment groups. In addition, body weight was determined every day.

### Faecal sampling

Mice in the DSS model (0%, 1%, or 1.5% DSS) were transferred from their home cage on d 0, d 5, and d 14 and mice from the stress model were transferred from their home cage on d 0, d 7, and d 10 for a period of 2 hours to a new cage containing LabSand (Coastline Global Inc., Palo Alto, United States) to collect a bulk sample of faecal pellets.

### Facial vein phlebotomy

Facial vein phlebotomy was performed in the respective cohorts (as specified in S1 Table) at d 0, d 5, and d 14, as described recently [27]. For this, mice were grabbed by the scruff of the neck to gently but firmly immobilize head, neck, and forelimbs without anaesthesia. The right lateral facial vein was then punctured with a 20-gauge needle. Phlebotomy was performed by the same trained and experienced person throughout the study. Approximately 15 μl of blood were collected with the Protein Saver Card (Whatman 903™, GE Healthcare Europe GmbH, Freiburg, Germany) to be stored as dried blood spots at room temperature for further analyses.

### Histology

A ‘Swiss roll’ was prepared from the colon, as described previously [55]. Colon samples were retrieved at d 14 and fixed in neutral buffered 4% formalin, processed routinely, embedded in paraffin, sectioned at 5–6 μm, and stained with hematoxylin and eosin. Histology slides were scored, as published recently, and by grading histopathologic lesions separately for the proximal and distal colon [54,56,57]. Scoring was performed blinded to sample identity/treatment group. Evaluated parameters included the presence of infiltrating inflammatory cells (severity and maximum extent); the intestinal architecture (epithelial and mucosal); the extent of edema, erosion, and ulceration; and the involved area. Each parameter was graded from 0 (no changes) to 4 (severe changes) in the proximal and distal colon sections, achieving a maximum score of 46.

### Statistics

Values are means ± standard error of the mean. All statistical analyses were performed using Graph-Pad Prism 5 and 6 software (La Jolla, California). All data were analysed with the Shapiro Wilk test for normal distribution. For parametric data, an unpaired *t* test with Welch’s correction in case of unequal variance or one-way analysis of variance (ANOVA) or repeated measure ANOVA was carried out. In case of ANOVA, Bartlett’s test was applied to check for homoscedasticity, and if the hypothesis of equal variance was rejected (*P* < 0.05), nonparametric methods were used. In inferential testing of multiple groups, *p*-values were adjusted for multiplicity during their individual posthoc testing procedure (Tukey test or Dunnett’s multiple comparison test). For nonparametric data, the Mann–Whitney or Wilcoxon test were performed to compare 2 groups. Other nonparametric data were analysed by the Friedman or Kruskal–Wallis test, both followed by Dunn’s multiple comparisons as posthoc test. *P* < 0.05 was considered significant. In all figures, * indicates *P* < 0.05, ** indicates *P* < 0.01, and *** indicates *P* < 0.001.

### K-means algorithm-based cluster analysis

To calculate clusters in order to assess and categorize severity, the R [58] software and unsupervised k-means clustering were used [58]. Regarding the general k-means clustering procedure, all data sets were retrieved from the experimental colitis group including standardized WR_20_ and body weight (BW). Both variables were used to calculate k-means clusters (701 × 2 data points out of *n* = 54 mice). Different conditions and days were pooled to include all possible states in one model. To calculate the cluster thresholds, the 701 × 2 data points were randomly divided into a training (80%) and a test set (20%). The training set was then used to calculate the thresholds. For stratification, this was repeated 100 times (with *q* = 0.8 × 701 = 561 permutations) at each run. Cluster thresholds were determined by calculating the median of the stratification data after filtering out extreme values; margins of 30% deviation in both directions from the median were allowed. The result was set as the global cluster threshold. This was repeated for each cluster, also resulting in 95% confidence borders (CBs; calculated by CB_95% CI_ = mean_thr_ ± 1.96 x SD(thr)/√561, with thr = all thresholds for each of the permutations and SD = standard deviation). The number of permutations was chosen to limit a potential overfitting of the resulting 95% CB and never exceeded the number of available data points per iteration. It was therefore considered to be fair. The 95% CBs reflect the randomness due to seeding during the clustering process and indicate a transition zone between the condition borders. Test samples in the confidence regions can be seen as ambiguous and cannot explicitly be allocated to either cluster.

For k-means optimization 2 methods, the scree plot and the Bayesian information criterion (BIC) were used, and for subsequent cluster stabilization analysis, seeding permutations were monitored. For scree plot analysis, the variation was analysed by the ‘within groups sum of squares’ at different cluster sizes. In the scree plot, three clusters were identified as the optimal size for a k-means clustering (S5A Fig). For validation, the R package Mclust [59] and the Mclust function were used to calculate the BIC. The BIC was calculated for 20 components (clusters) in 14 multivariate models. All multivariate models except EII and VII had a maximum BIC at three clusters. However, as EII and VII are both spherical models but the analysed data are rather diagonal and ellipsoidal, these models were not included in the determination of the optimal cluster size (S5B Fig). Cluster stability was also monitored by permutation analysis. For this, the median of 100 samples with 561 permutations, each with different seeding positions, were analysed. The median upper threshold at random seeding over 100 iterations was WR_20_ = 87.37% and the lower median threshold WR_20_ = 50.16%. Out of 100 iterations, no cluster showed outliers above or below 1% deviation from the median. Therefore, the median cluster thresholds from the random permutations can be considered stable (S5C Fig).

## Supporting information

S1 Table Experimental set up. After a 2-week habituation to the animal room, animals were divided into treatment and control groups by applying a random selection procedure (drawing lots). A 2-week adaption phase to wheel running was chosen.
(DOCX)

S1 Data Underlying numerical data. Excel spreadsheet containing, in separate sheets, the underlying numerical data for Figs 1A, 1B, 1C, 1D, 1E, 2B, 2C, S1A, S1B, S2A, S2B, S2C, S3A, S3B, S3C and S4M. The underlying numerical data of Figs 1F, 1G, 1H and 1I, as well as 2D, 2E, 2F and 2G are provided in the corresponding sheet of Fig 1E.
(XLSX)

S1 Fig Adaptation to the running wheel. (a) Monitoring of WR_20_ and (b) Vmax_20_ in B6 mice during the 14-day adaption phase (*n* = 52). ***P < 0.001 compared to d 1 of monitoring by Friedman test followed by Dunn’s multiple comparison test. The underlying numerical data are provided in S1 Data. B6, C57BL/6J; Vmax_20,_ maximum velocity during 20 hours/day; WR_20,_ wheel rotations during 20 hours/day
(TIF)

S2 Fig Clinical scoring during colitis and restraint stress. (a) Clinical score determined in DSS-treated and control mice and (b) DSS-treated and control mice additionally submitted to facial vein phlebotomy (see S1 Table for groups and *n* values). (c) Clinical scoring in mice undergoing repeated restraint stress (*n* = 8). *P < 0.05, **P < 0.01, and ***P < 0.001; colours indicate comparison between respective groups: medium grey between 0% and 1%, black between 0% and 1.5%, and light grey between 1% and 1.5% (a, b Kruskal–Wallis test followed by Dunn’s multiple comparison test, c Wilcoxon signed rank test) and underlined asterisks indicate the comparison to baseline levels within a group (Friedman test followed by Dunn´s multiple comparison test). The underlying numerical data are provided in S1 Data. DSS, dextran sulfate sodium
(TIF)

S3 Fig Assessment of running velocities (Vmax_20_). (a) Monitoring of Vmax_20_ in DSS-treated and control mice and (b) DSS-treated and control mice submitted to facial vein phlebotomy (for *n* values see S1 Table); colours indicate comparison between respective groups: medium grey between 0% and 1%, black between 0% and 1.5%, and light grey between 1% and 1.5%. (c) Vmax_20_ in mice undergoing repeated restraint stress (*n* = 8). *P < 0.05, **P < 0.01, and ***P < 0.001 comparison between groups (a, b one-way ANOVA, subsequent Tukey posthoc test or Kruskal–Wallis test followed by Dunn’s multiple comparison test, c unpaired *t* test with Welch’s correction in case of unequal variance or Mann–Whitney test) and underlined asterisks indicate the comparison to baseline levels within a group (repeated measure ANOVA followed by Dunnett’s posthoc test or Friedman test followed by Dunn’s multiple comparison test). The underlying numerical data are provided in S1 Data. DSS, dextran sulfate sodium
(TIF)

S4 Fig Colon histology. (a–l) Histological analysis corroborates aggravated colitis course. Colon tissue obtained from B6 mice treated with 0% (a–b), 1% (c–d) and 1.5% (e–f) DSS, respectively. Histological alterations were not detected in the 0% DSS treatment groups with or without blood sampling (a–b, g–h). All mice treated with DSS developed a mild to profound colitis characterized by mixed cell infiltrates, abnormal crypt architecture, edema, and erosions (d, f). Statistically significant differences in the histological score were detected between untreated and 1.5% DSS treated mice (m); mice receiving 1% DSS displayed intermediate scores (m). Blood sampling by facial vein phlebotomy led to enhanced histological scores in mice receiving 1% and 1.5% DSS (i–j, k–l). Intestinal alterations were more pronounced and characterized by mixed cell infiltration, abnormal crypt architecture, goblet cell and epithelial loss, ulcerations, and transmural inflammatory processes (j, l). Original magnification 5x and 10x. (m) Histological score quantifying severity of colitis (Median ± min/max; for *n* values see S1 Table and S1 Data, *P < 0.05 and **P < 0.01 compared to other groups by one-way ANOVA followed by Tukeys posthoc test or Kruskal–Wallis test followed by Dunn’s multiple comparison test). The underlying numerical data are provided in S1 Data. B6, C57BL/6J; DSS, dextran sulfate sodium
(TIF)

S5 Fig Scree plot analysis, Bayesian information criterion, and seeding permutation for clustering. (a) Determination of the cluster number by scree plot analysis. Within the scree plot method, three clusters were identified as the optimal size for k-means clustering (dashed line). (b) Utilization of the BIC to validate the number of clusters. All multivariate models except EII and VII had a maximum BIC at three clusters (dashed line). (c) Monitoring of cluster stability by seeding permutations. The median upper threshold at random seeding over 100 iterations was WR_20_ = 87.37% (95% CB [83.75; 90.39]), the lower median threshold WR_20_ = 50.16% (95% CB [46.43; 53.57]). BIC, Bayesian information criterion; CB, confidence border; WR_20,_ wheel rotations during 20 hours/day
(TIF)

## Abbreviations

3Rs: replace, reduce, and refine
B6: C57BL/6J
BIC: Bayesian information criterion
CB: confidence border
DSS: dextran sulfate sodium
EU: European Union
Vmax_20_: maximum velocity during 20 hours/day
VWR: voluntary wheel running
WR_20_: wheel rotations during 20 hours/day
