# Supplementary material for: Running in the wheel: Defining individual severity levels in mice
Source: PLoS Biol. 2018 Oct 18;16(10):e2006159. doi: 10.1371/journal.pbio.2006159 (PMC6193607; doi:10.1371/journal.pbio.2006159)
Supplement: S1 Table — After a 2-week habituation to the animal room, animals were divided into treatment and control groups by applying a random selection procedure (drawing lots). A 2-week adaption phase to wheel running was chosen. (DOCX) [file pbio.2006159.s001.docx]

| Treatment | Phlebotomy | Faecal sampling | Wheel access | n= |
| --- | --- | --- | --- | --- |
| DSS 0% (control) | - | d0, d5, d14 | + | 7 |
| DSS 1%  d1-d5 | - | d0, d5, d14 | + | 8 |
| DSS 1.5%  d1-d5 | - | d0, d5, d14 | + | 8 |
| DSS 0% (control) | d0, d5, d14 | d0, d5, d14 | + | 8 |
| DSS 1%  d1-d5 | d0, d5, d14 | d0, d5, d14 | + | 14 |
| DSS 1.5%  d1-d5 | d0, d5, d14 | d0, d5, d14 | + | 9 |
| control | - | d0, d7, d10 | + | 8 |
| restraint stress  d1-d10 | - | d0, d7, d10 | + | 8 |

**S1 Table**
