# Supplementary figures and images for: Running in the wheel: Defining individual severity levels in mice
Source: PLoS Biol. 2018 Oct 18;16(10):e2006159. doi: 10.1371/journal.pbio.2006159 (PMC6193607; doi:10.1371/journal.pbio.2006159)

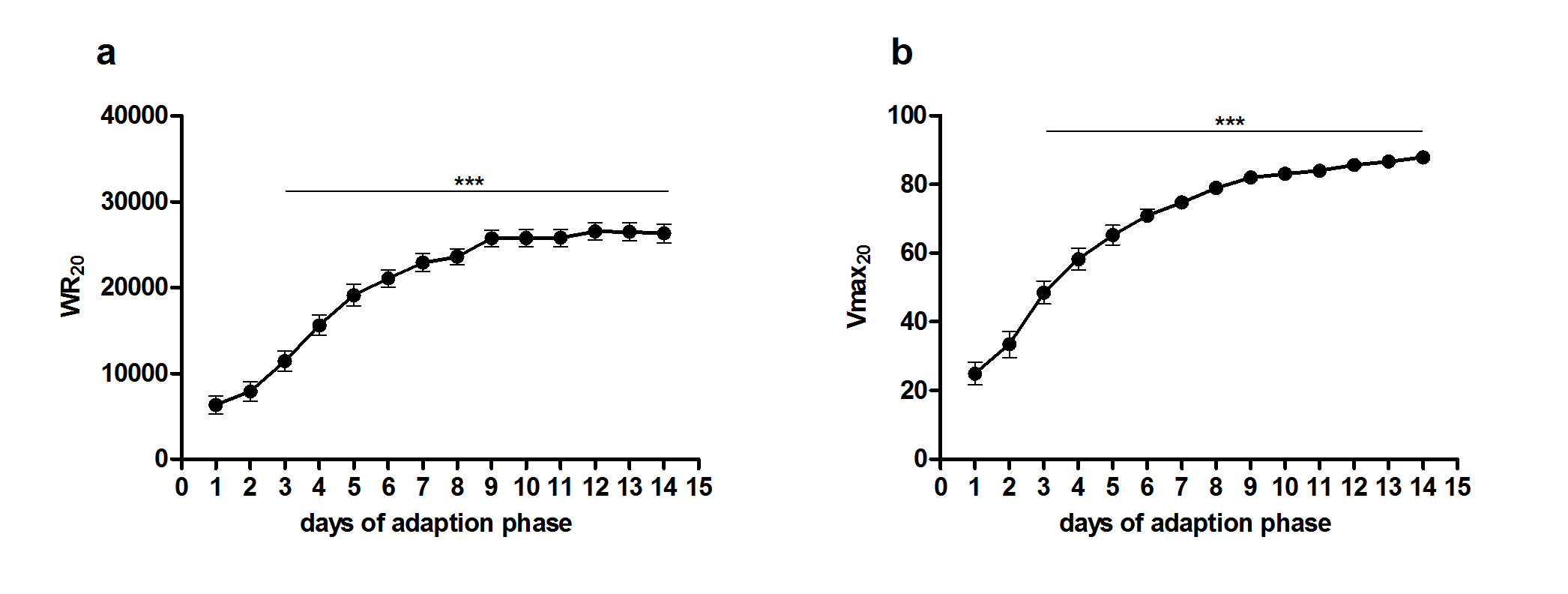

Supplement: S1 Fig — (a) Monitoring of WR20 and (b) Vmax20 in B6 mice during the 14-day adaption phase (n = 52). ***P < 0.001 compared to d 1 of monitoring by Friedman test followed by Dunn’s multiple comparison test. The underlying numerical data are provided in S1 Data. B6, C57BL/6J; Vmax20, maximum velocity during 20 hours/day; WR20, wheel rotations during 20 hours/day (TIF) [file pbio.2006159.s003.tif]

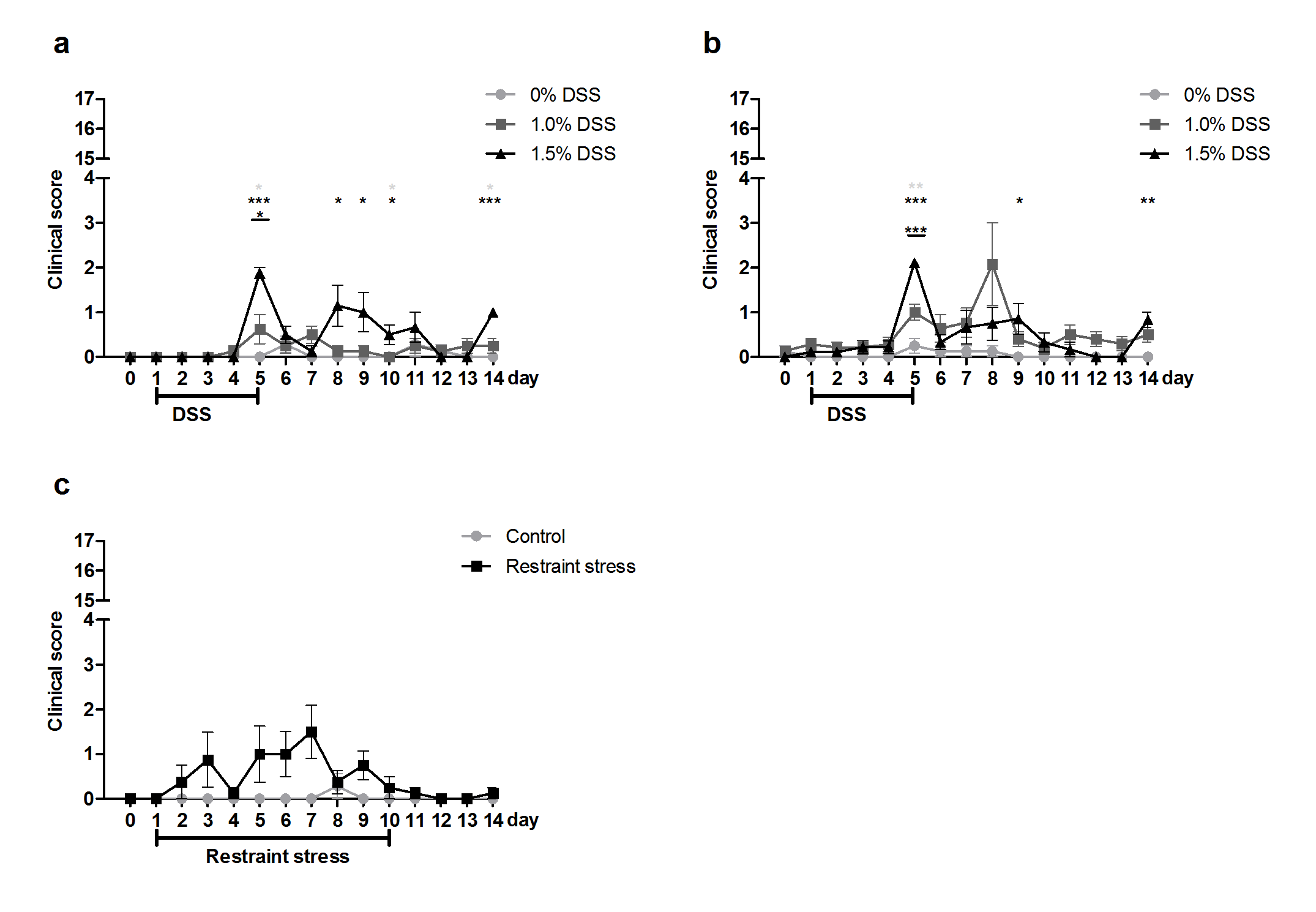

Supplement: S2 Fig — (a) Clinical score determined in DSS-treated and control mice and (b) DSS-treated and control mice additionally submitted to facial vein phlebotomy (see S1 Table for groups and n values). (c) Clinical scoring in mice undergoing repeated restraint stress (n = 8). *P < 0.05, **P < 0.01, and ***P < 0.001; colours indicate comparison between respective groups: medium grey between 0% and 1%, black between 0% and 1.5%, and light grey between 1% and 1.5% (a, b Kruskal–Wallis test followed by Dunn’s multiple comparison test, c Wilcoxon signed rank test) and underlined asterisks indicate the comparison to baseline levels within a group (Friedman test followed by Dunn´s multiple comparison test). The underlying numerical data are provided in S1 Data. DSS, dextran sulfate sodium (TIF) [file pbio.2006159.s004.tif]

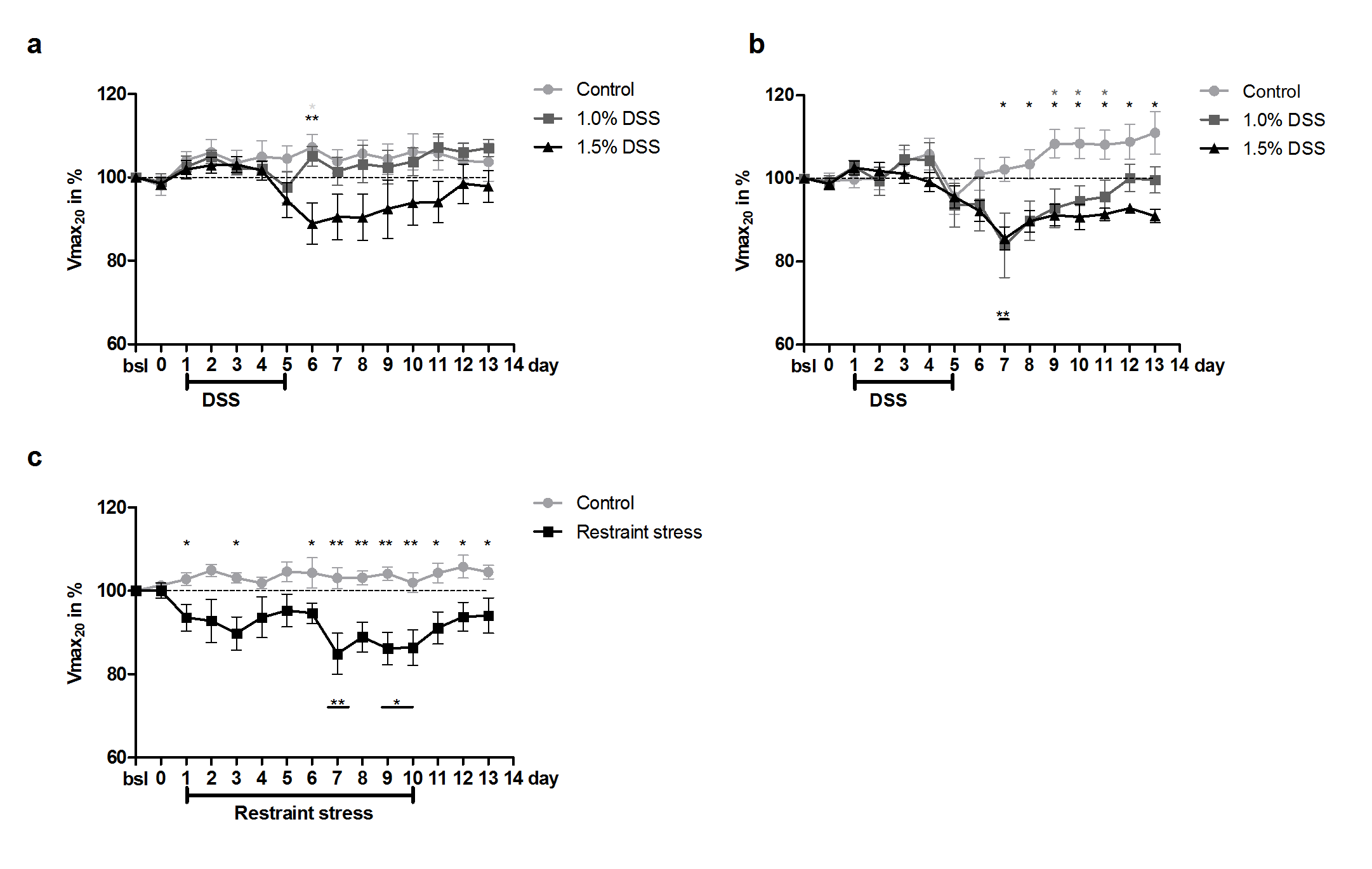

Supplement: S3 Fig — (a) Monitoring of Vmax20 in DSS-treated and control mice and (b) DSS-treated and control mice submitted to facial vein phlebotomy (for n values see S1 Table); colours indicate comparison between respective groups: medium grey between 0% and 1%, black between 0% and 1.5%, and light grey between 1% and 1.5%. (c) Vmax20 in mice undergoing repeated restraint stress (n = 8). *P < 0.05, **P < 0.01, and ***P < 0.001 comparison between groups (a, b one-way ANOVA, subsequent Tukey posthoc test or Kruskal–Wallis test followed by Dunn’s multiple comparison test, c unpaired t test with Welch’s correction in case of unequal variance or Mann–Whitney test) and underlined asterisks indicate the comparison to baseline levels within a group (repeated measure ANOVA followed by Dunnett’s posthoc test or Friedman test followed by Dunn’s multiple comparison test). The underlying numerical data are provided in S1 Data. DSS, dextran sulfate sodium (TIF) [file pbio.2006159.s005.tif]

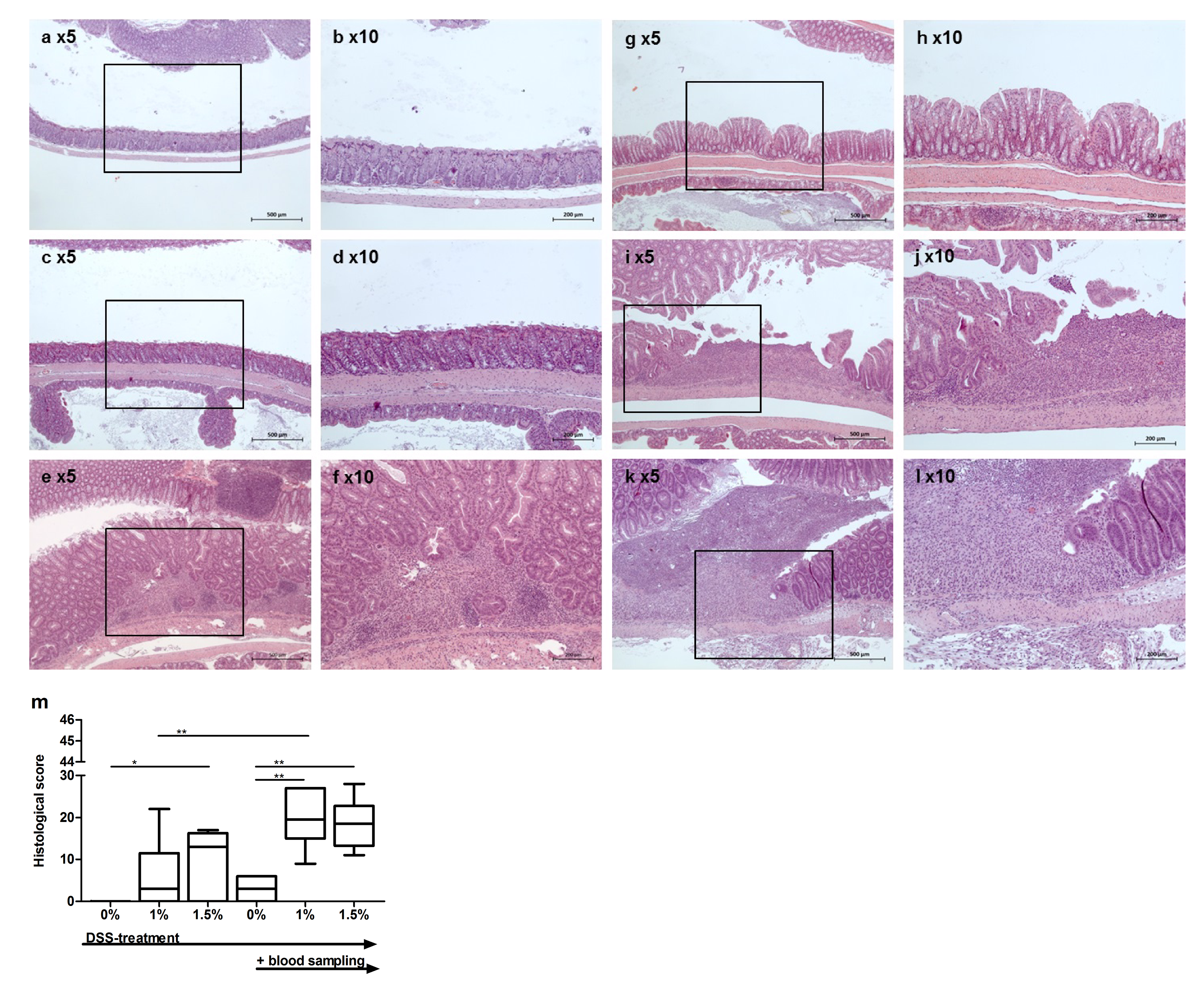

Supplement: S4 Fig — (a–l) Histological analysis corroborates aggravated colitis course. Colon tissue obtained from B6 mice treated with 0% (a–b), 1% (c–d) and 1.5% (e–f) DSS, respectively. Histological alterations were not detected in the 0% DSS treatment groups with or without blood sampling (a–b, g–h). All mice treated with DSS developed a mild to profound colitis characterized by mixed cell infiltrates, abnormal crypt architecture, edema, and erosions (d, f). Statistically significant differences in the histological score were detected between untreated and 1.5% DSS treated mice (m); mice receiving 1% DSS displayed intermediate scores (m). Blood sampling by facial vein phlebotomy led to enhanced histological scores in mice receiving 1% and 1.5% DSS (i–j, k–l). Intestinal alterations were more pronounced and characterized by mixed cell infiltration, abnormal crypt architecture, goblet cell and epithelial loss, ulcerations, and transmural inflammatory processes (j, l). Original magnification 5x and 10x. (m) Histological score quantifying severity of colitis (Median ± min/max; for n values see S1 Table and S1 Data, *P < 0.05 and **P < 0.01 compared to other groups by one-way ANOVA followed by Tukeys posthoc test or Kruskal–Wallis test followed by Dunn’s multiple comparison test). The underlying numerical data are provided in S1 Data. B6, C57BL/6J; DSS, dextran sulfate sodium (TIF) [file pbio.2006159.s006.tif]

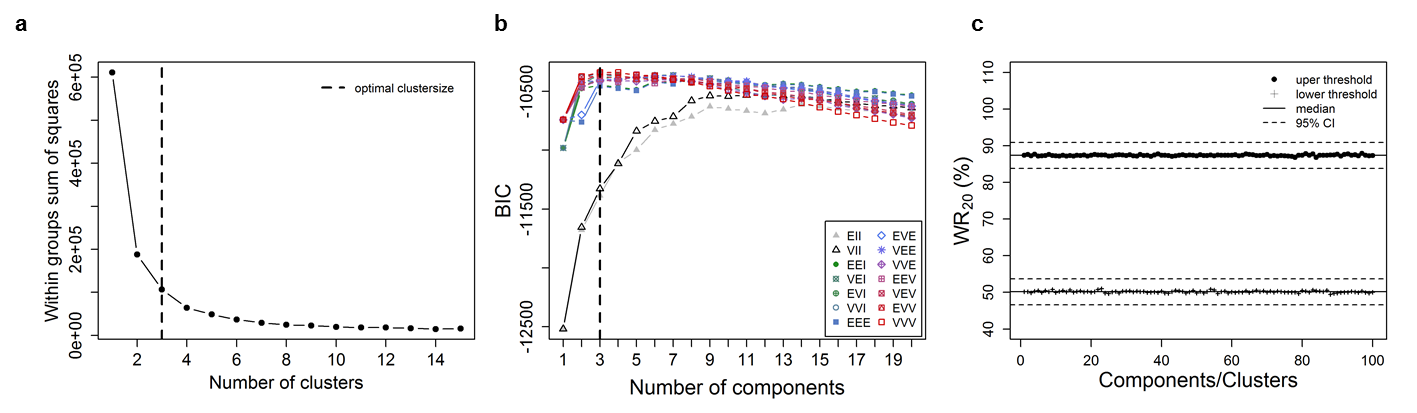

Supplement: S5 Fig — (a) Determination of the cluster number by scree plot analysis. Within the scree plot method, three clusters were identified as the optimal size for k-means clustering (dashed line). (b) Utilization of the BIC to validate the number of clusters. All multivariate models except EII and VII had a maximum BIC at three clusters (dashed line). (c) Monitoring of cluster stability by seeding permutations. The median upper threshold at random seeding over 100 iterations was WR20 = 87.37% (95% CB [83.75; 90.39]), the lower median threshold WR20 = 50.16% (95% CB [46.43; 53.57]). BIC, Bayesian information criterion; CB, confidence border; WR20, wheel rotations during 20 hours/day (TIF) [file pbio.2006159.s007.tif]
